# Evaluating the potential outcomes of pharmacist-led activities in the Australian general practice setting: a prospective observational study

**DOI:** 10.1007/s11096-023-01604-x

**Published:** 2023-06-03

**Authors:** Thilini Sudeshika, Louise S. Deeks, Mark Naunton, Gregory M. Peterson, Sam Kosari

**Affiliations:** 1 Discipline of Pharmacy, Faculty of Health, University of Canberra, Bruce, ACT 2617 Australia; 2 Department of Pharmacy, Faculty of Allied Health Sciences, University of Peradeniya, Peradeniya, 20400 Sri Lanka; 3 School of Pharmacy and Pharmacology, University of Tasmania, Hobart, TAS 7005 Australia

**Keywords:** Clinical activities, Clinical impact, Economic impact, Evaluation, General practice pharmacist, Medicines, Organisational impact

## Abstract

**Background:**

Pharmacists have been co-located in general practice teams to support the quality use of medicines and optimise patient health outcomes. Evidence of the impact of pharmacist-led activities in Australian general practices is sparse.

**Aim:**

This study aimed to evaluate the potential outcomes of pharmacist-led activities in Australian general practices.

**Method:**

A prospective observational study was conducted in eight general practices in the Australian Capital Territory, where each general practice employed a pharmacist on a part-time basis for 18 months. A recommended, but flexible, list of activities was provided for pharmacists. Descriptive information on general practice pharmacist-led activities, collected with an online diary, was analysed. The potential clinical, economic, and organisational impact of pharmacist-led clinical activities was evaluated using the CLinical Economic Organisational (CLEO) tool, with a modified economic dimension.

**Results:**

Nine pharmacists reported 4290 activities over 3918.5 work hours in general practice. Medication management services were the primary clinical activity of pharmacists. In medication reviews, 75% of the pharmacists’ recommendations were fully accepted by general practitioners. Conducting clinical audits, updating patients’ medical records, and providing information to patients and staff were other major activities of pharmacists. Of 2419 clinical activities, around 50% had the potential for a moderate or major positive clinical impact on patients. Sixty-three per cent of activities had the potential to decrease healthcare costs. Almost all the pharmacist-led clinical activities had a positive organisational impact.

**Conclusion:**

Most pharmacist-led clinical activities in general practice had the potential for a positive impact on patients and reduction in healthcare costs, supporting the expansion of this model in Australia.

**Supplementary Information:**

The online version contains supplementary material available at 10.1007/s11096-023-01604-x.

## Impact statements

- Pharmacists can provide numerous services to improve the quality of care in the general practice setting by working in a team environment.
- Most general practice pharmacist-led clinical activities had the potential to improve patient care and decrease healthcare costs.

## Introduction

General practices represent an important primary care service that assists the community to manage medical problems and reduce hospital admissions through prevention and early interventions [1]. However, general practices face challenges due to the escalating demand for providing high-quality patient care. The increasing older population, growing burden of chronic diseases, workforce shortages, and reduced general practitioners’ (GPs’) working hours are placing pressure on general practices worldwide [2–4]. This suggests the need for team-based care models in general practices to support GPs and improve patient outcomes [5].

Pharmacists have a role to ensure appropriate, safe, and effective drug therapy. Models incorporating pharmacists in general medical practices have been developed to optimise medication use [6]. General practice pharmacists can identify, resolve, prevent, and monitor medication-related problems [7, 8]. In addition to patient-specific medication management services, general practice pharmacists may provide various services, such as quality assurance activities, requesting laboratory tests, providing education to staff and patients, and updating clinical documentation [9, 10]. Incorporating pharmacists in general practices has been studied in many countries. There is a growing international evidence base demonstrating that general practice-based pharmacists can improve medication use through individual patient assessments, population-based interventions and implementing system-level practice enhancements [11–15].

Pharmacists were introduced in Australian general practices in the early 2010s. However, the uptake of pharmacist inclusion in general practice teams has been very slow across the country. There have been several studies conducted in Australia related to pharmacists working in general practices, with limited investigation into pharmacist-led clinical activities, their impact and patient outcomes [7, 10, 16–19]. There is a need for more research to describe pharmacist-led activities and the impact of those activities in general practices [20].

In the Australian Capital Territory (ACT), a pilot study was conducted to explore the inclusion of pharmacists in general practices through funds from the Capital Health Network (CHN: Primary Health Care Network in the ACT). This pilot study reported general practice pharmacists can engage in a range of activities to support GPs [10]. The study showed that general practice pharmacists can perform medication reviews, provide patient and staff education, assist in the management of asthma, conduct smoking cessation programs, undertake clinical audits, recommend de-prescribing, and contribute to Medicare Benefits Schedule (MBS) activities that generate income for the general practice [10, 21–23]. The promising findings of this pilot study informed the expansion of a larger project in multiple locations in the ACT from 2019 to 2021 [24].

### Aim

The aim of this study was to evaluate the potential outcomes of pharmacists-led activities in the general practices.

### Ethics approval

This study was approved by the Human Research Ethics Committee at the University of Canberra on 20 December 2018 (Ref: HREC 15-235).

## Method

### Study design and setting

A prospective observational study was conducted in eight general practices in the ACT to evaluate the activities of general practice pharmacists.

### Participants and recruitment

The eight general practices were identified by the funder via an expression of interest. The practices were selected to reflect a mix of government-subsidised (“bulk-billing”) and private billing funded practices, at locations across the ACT. The pharmacists (n = 9) were recruited by the general practices between 2019 and 2020. In one general practice, a pharmacist left the project within 5 months and another pharmacist was employed for the remaining time of the contract. The researchers were not involved in the recruitment of general practices or employment of pharmacists.

### Intervention

Pharmacists were employed on a part-time basis (15 h per week) for 18 months in the eight general practice sites to provide non-dispensing services. These pharmacists did not have prior experience with the general practice pharmacist role in Australia. A recommended, but flexible, list of activities for pharmacists was developed by the research team based on the previous pilot study and international literature [10, 11, 13, 14] (Table 1). These activities often involved communication with other health professionals both within and external to the practice, including community pharmacies, hospitals, residential aged care facilities (RACFs), and community out-reach services. Each pharmacist was allocated an eight-hour mentorship from a pharmacist who had worked in general practice.

**Table 1:** Recommended activities for the pharmacists in general practices

Activity	Expected outcome
Medication review	Improve medication safety with a focus on deprescribing
Antimicrobial stewardship	Prevent overuse of antimicrobial agents and development of resistance
Clinical audits	Improve quality of prescribing at practice level
Point-of-care-testing	Monitor existing conditions and identify undiagnosed diseases
Management of asthma	Improve asthma management and control patients’ symptoms
Providing education to staff	Assist general practice staff to improve knowledge on medications, drug interactions, adverse effects, guidelines, correct use of medical devices
Diabetes education	Improve diabetes care management through pharmacological and non-pharmacological interventions
Updating medical records	Drug allergy/adverse drug reaction information
Transitions of care	Accurate medicine reconciliation after hospital discharge
Smoking cessation	Support people to quit smoking through pharmacological and non-pharmacological interventions
Vaccination	Complement existing immunisation services by administering vaccines
Collaboration with external services/healthcare professionals	Act as a conduit between the general practice and other external services e.g. community pharmacies, hospitals, RACF

### Data collection

Pharmacists’ daily activities were reported using an online electronic diary (Google Forms Online), in which activities were categorised according to the list in Table 1. Miscellaneous activities, such as service development (meeting, planning, booking appointments), professional development and other activities, were categorised separately. In the activity diary, pharmacists were asked to record the estimated time taken to conduct each activity, whether they contacted the patient, details and outcome of the activity, whether the activity saved time for GPs, and the type of claim or MBS item (government-subsidised medical activity) which generated income for the practice and their estimated contribution to the income generated where applicable. One of the researchers (LSD) communicated with the pharmacists monthly to discuss any queries related to data collection. Additionally, pharmacists, practice managers and the research team were invited to participate in quarterly meetings organised by the funder to discuss study activities. Pharmacists’ activities were collected from February 2019 to April 2021.

### Data analysis

#### Coding pharmacists’ activities

Data were de-identified prior to analysis. Pharmacist-led activities were analysed by using an evolving coding system. This system consisted of major codes and sub-codes; major codes were the recommended pharmacist activities (Table 1). Sub-codes included type of activity, referral to the activity, time and contribution to the activity, collaboration, and the outcome of the activity. During data analysis, other activities that did not belong to the above major codes or sub-codes were coded separately. To prevent multiple codes, the activities were categorised according to the definitions provided in Supplemental file 1. The activities were coded independently by two researchers (TS, LSD). Any discrepancies in coding were resolved by a third researcher (SK). Generated income from MBS claimable activities was calculated through the pharmacists’ contribution and AU$ values of MBS items identified at the time of the study [23, 25]. Descriptive statistics were used where applicable to provide an overview of the details of all activities conducted by pharmacists. Categorical measures were presented as the frequencies and percentages, while continuous measures were presented as the mean and standard deviation. The data were analysed using Statistical Package for the Social Sciences (SPSS ver. 27 IBM Corp, Armonk, New York, USA).

#### Evaluating the potential outcomes of pharmacist-led clinical activities

Pharmacist-led clinical activities in general practice were evaluated by using the CLinical Economic Organisational (CLEO) tool [26]. The multidimensional CLEO tool was based on a review of models and tools to assess pharmacist interventions [27]. This tool has been utilised to explore the impact of pharmacist activities using routine data in the hospital setting [28]. The economic dimension of the CLEO tool was modified to be utilised in the general practice setting (Supplemental file 2).

To test the modified CLEO tool, an assessment was conducted for pharmacist-led activities (n = 33) by selecting the first three activities in the activity diary reflecting each of the 11 different pharmacists’ clinical activities focused on in this study. These pharmacists’ clinical activities included medication reviews, antimicrobial stewardship (AMS), clinical audits, point of care testing (POCT), asthma care, providing education to staff related to medication management of patients, diabetes education, updating medical records, transitions of care, smoking cessation, and vaccination. Pharmacists’ activities related to collaboration with external services, providing basic or general information to staff (e.g. medicine shortages and recalls), service development, and miscellaneous activities were excluded from the evaluation. Inter-rater reliability was tested by checking the intra-class correlation coefficient (ICC_A,1_) in the assessment [26, 29]. To check inter-rater reliability, the selected pharmacists’ clinical activities were graded independently by three raters (TS, LSD, SK) [30]. The inter-rater reliability for the modified CLEO was good for the clinical (ICC_A,1_ = 0.77) and economic (ICC_A,1_ = 0.81) dimensions, and excellent for the organisational dimension (ICC_A,1_ = 0.99).

Then, two researchers (TS, LSD) independently applied the CLEO criteria to 10% of the pharmacist-led clinical activities reported through the activity diary. Of these activities, discrepancies for only 18 activities (i.e. agreement was more than 90%) were resolved by a third researcher (SK). Based on the analysis, guidance was developed and modified to rate the remaining 90% of activities. Finally, a single investigator (TS) evaluated the remaining 90% of pharmacist-led clinical activities. The modified CLEO tool has been provided in Supplemental file 2.

## Results

### Description of pharmacist-led activities

Nine pharmacists reported 4290 activities over 3918.5 work hours. After removing 20 duplicate entries, 4270 activities were included in the analysis. There were 1912 (45%) records of patient-facing activities and 2358 (55%) records of non-patient facing activities. The most frequently reported patient-facing activity by pharmacists was the conduct of medication reviews (932; 22%). In medication reviews, 75% of the pharmacists’ recommendations were fully accepted and 10% were partially accepted by GPs (Table 2). The most frequently reported non-patient facing activities were service development (1111; 26%) and collaboration with external services (360; 8%). Overall, 2452 (57%) pharmacists’ activities were directly related to the quality use of medicines.

**Table 2:** Description of pharmacists’ activities in general practice

Activity	Reported by: pharmacists n (%)	Number of activities n (%)	Time spent per activity (minutes) mean ± SD	Estimated time saved for GPs per activity (minutes) mean ± SD	Description/outcomes
Medication review	9 (100)	932 (22)	42.6 ± 25.0	33.5 ± 24.5	Pharmacists’ interventions*: providing education (n = 735, 79%), monitoring the condition (n = 496, 53%), dose adjustments (n = 262, 28%), deprescribing (n = 252, 27%), referrals (n = 223, 24%), initiation of new therapy (n = 209, 22%) Acceptance of pharmacists’ recommendations by GPs: accepted (n = 698, 75%), partly accepted (n = 97, 10%), unknown (n = 96, 10%), no action required by GPs (n = 25, 3%), not accepted (n = 18, 2%)
Antimicrobial stewardship	7 (78)	24 (1)	84.4 ± 70.7	2.9 ± 8.2	Collecting data/audit (n = 16, 67%), providing education (n = 8, 33%)
Clinical audits	9 (100)	294 (7)	84.3 ± 77.6	N/A	Clinical audits related to: diabetes and no record of HbA1c (n = 40, 14%); inappropriate use of proton pump inhibitors (high dose [> 40 mg/day esomeprazole equivalent] with no relevant indication for > 4 weeks) (n = 39, 13%); people who were eligible for health checks, home medication reviews, and case conferences (n = 32, 11%); people who were eligible but not received vaccines (n = 27, 9%); anticoagulation therapy (AF patients at high risk of stroke/patients who had been prescribed dual antiplatelet therapy beyond indicated time) (n = 22, 7%); polypharmacy ( patients on 5 or more medicines) (n = 22, 7%); CKD patients who required dose adjustments (n = 19, 6%); medicines use in asthma/COPD (n = 15, 5%); opioids use in non-cancer pain (n = 15, 5%); osteoporosis (patients at high risk due to long-term use of systemic corticosteroids/had not been prescribed anti-osteoporosis medication for minimal trauma fractures/timely administration of denosumab) (n = 13, 4%); antipsychotics prescribed without monitoring BP, BGL, weight, cholesterol (n = 10, 3%); angiotensin converting enzyme inhibitors or angiotensin receptor blockers use in heart failure (n = 8, 3%); patients who eligible for vitamin D treatment (n = 8, 3%); inappropriate use of NSAIDs (older age, heart failure) (n = 7, 2%); patient records required updating (allergies/other socio-demographic details) (n = 7, 2%); other (n = 10, 3%) (contraception status of females prescribed valproate, product alerts, Hepatitis C treatment)
Point-of-care-testing (POCT)	6 (67)	64 (2)	20.8 ± 19.3	N/A	Pharmacist-led POCT*—The number of patients screened for: blood pressure (n = 38, 59%), asthma/COPD (n = 27, 42%), blood glucose level (n = 21, 33%), AF (n = 7, 11%), other (n = 7, 11%) Outcomes: case identification (n = 26, 41%), no action required (n = 16, 25%), further reviews/referrals (n = 8, 13%), treatment initiated/added a medicine (n = 5, 8%), ceased medicines (n = 3, 5%), dose adjustments (n = 2, 3%), outcome not reported (n = 4, 6%)
Asthma care	7 (78)	133 (3)	30.7 ± 9.3	26.3 ± 8.9	Pharmacists’ interventions*: Providing education—inhaler counselling (n = 59, 44%), lifestyle modifications (n = 43, 32%); recommended step-up of asthma prevention treatment 44 (33%), recommended step-down of asthma prevention treatment 14 (11%), developing asthma action plan (n = 39, 29%), changed medicines/device (n = 17, 13%)
Providing education to general practice staff	9 (100)	301 (7)	32.5 ± 24.1	27.8 ± 19.5	Providing medicine information related to patients’ therapy (n = 175, 58%) Providing general education on guidelines, medicine availability, updates from Therapeutic Goods Administration, changes to Pharmaceutical Benefits Scheme (n = 126, 42%)
Diabetes education (including as a CDE)	2 (22)	385 (9)	36.8 ± 16.5	20.5 ± 13.2	Pharmacists’ interventions*: providing education on diet/lifestyle modification (n = 201, 52%), self-blood glucose monitoring (n = 137, 36%), insulin storage/administration (n = 107, 28%); medication adjustments and providing education on medicines (n = 170, 44%); referrals (n = 6, 2%)
Updating medical records	4 (44)	72 (2)	113.9 ± 109.7	N/A	Pharmacists updated 2519 patient records, including updating the allergy status (n = 1102, 44%); updating diagnosis, and social/lifestyle (n = 843, 33%); updating the medication list (n = 384, 15%); updating ADR (n = 190, 8%) for patients
Transitions of care	7 (78)	160 (4)	29.9 ± 14.5	29.4 ± 13.9	Pharmacists’ interventions: medicine reconciliation (n = 89, 56%), providing education (n = 51, 32%), updating records for (n = 20, 13%) patients
Smoking cessation	6 (67)	161 (4)	34.9 ± 12.3	30.2 ± 7.0	Of 161 smoking cessation sessions conducted by pharmacists, 86 (53%) were the first-time appointments and 75 (47%) were follow-up sessions. Of 86 patients, 36 (42%) patients were attempting to quit smoking, 21 (24%) patients reduced smoking, 14 (16%) quit smoking, and 4 (5%) did not quit smoking
Vaccination	5 (56)	52 (1)	35.1 ± 39.3	N/A	Of 126 patients, the influenza vaccine was administered for 110 (87%), and the COVID-19 vaccine for 16 (13%) patients
Collaboration and communication with external services/healthcare professionals	9 (100)	360 (8)	46.0 ± 50.6	28.0 ± 31.0	Activities related to collaboration with a community pharmacy included: queries related to adherence/prescriptions/patient records (n = 140, 39%); opioid maintenance treatment delivery (n = 74, 21%); obtaining medicine availability information (n = 71, 20%); referrals (HMR, dose aid administration/sleep apnoea) (n = 36, 10%); obtaining dispensing history (n = 23, 6%); drug disposal (n = 5, 1%) Activities related to collaboration with other services/healthcare professionals (n = 11, 3%)
Service development	9 (100)	1111 (26)	73.2 ± 72.0	N/A	Planning for new service implementation (n = 290, 26%), meeting others about the service (n = 291, 26%), training for the role/continuous professional development (n = 288, 26%), administrative work (n = 127, 11%), booking appointments for patients (n = 115, 10%)
Miscellaneous activities	9 (100)	221 (5)	45.3 ± 36.8	N/A	Doctors’ bag check/stock management or expiry check of the medicines in the general practice (n = 86, 39%); Project-related activities, medical assistance, and other activities (n = 135, 61%)

Due to rounding, numbers may not always add to 100%

*HMR* Home medicines review, *CDE* Credentialed diabetes educator, *ADR* Adverse drug reaction, *AF* Atrial fibrillation, *CKD* Chronic kidney disease, *BP* Blood pressure, *BGL* Blood glucose level, *COPD* Chronic obstructive pulmonary disease, *NSAIDs* Non-steroidal anti-inflammatory drugs

*These activities may include more than one pharmacist intervention. Therefore, numbers may not always add to 100%

Pharmacists supported GPs to generate income for the general practice through contribution to MBS claimable activities in medication reviews, POCT, asthma care, diabetes education, transitions of care, and smoking cessation (Table 3).

**Table 3:** Pharmacists’ contribution to generate income through Medicine Benefits Schedule (MBS) items

Activity	Claimed MBS items	Generated income per pharmacist AU$
Medication review	Health assessment 707	6408
	GP management plan 721	
	Team care arrangement 723	
	Review of GP management plan or team care arrangement 732	
	Multidisciplinary case conferences 735, 747, 739, 750	
	Home medicines review 900	
Point-of-care-testing	Contribution to spirometry 11,505	85
Asthma care	Asthma cycle of care: 265, 2546, 2552, 2558	1088
	GP management plan 721	
	Multidisciplinary case conference 735	
Diabetes education	Diabetes cycle of care 2525	8090
	Diabetes education 10,951	
	Multidisciplinary case conference 735, 739	
	GP management plan 721	
	Team care arrangement 723	
	Allied health COVID telehealth 93,013	
Transitions of care	Multidisciplinary case conference 735	96
	GP management plan 721	
	Allied health COVID telehealth 93,013	
Smoking cessation	Team care arrangement 732	37
	GP mental health treatment plan 2713	

### Potential outcomes of pharmacist-led clinical activities

After excluding the pharmacists’ activities related to collaboration with external services (n = 360), providing general education to staff on guidelines and medicine availability etc. (n = 126), service development (n = 1111), miscellaneous tasks (n = 221) and activities utilised to test the modified CLEO tool (n = 33), 2419 clinical activities conducted by pharmacists were included in the assessment. The clinical activities were related to medication reviews, AMS, clinical audits, POCT, asthma care, providing education to staff, diabetes education, updating medical records, transitions of care, smoking cessation, and vaccination. The potential clinical impact was considered major for 787 (32.5%) clinical activities and moderate for 407 (16.8%) activities (Table 4). Overall, 1528 (63.2%) clinical activities potentially decreased costs to the healthcare system. The organisational impact was positive for almost all the pharmacist-led clinical activities (2367, 97.9%). Examples of case scenarios have been provided in Supplemental file 3.

**Table 4:** Impact of general practice pharmacists’ clinical activities assessed as per the adapted CLEO tool

Dimension	Definition	Impact	Number of activities (%)
Clinical	The PI can lead to adverse outcomes on clinical status, knowledge, satisfaction, patient adherence and/or quality of life of the patient	Negative	8 (0.3)
	The PI can have no influence on the patient regarding the clinical status, knowledge, satisfaction, patient adherence and or quality of life of the patient	Null	189 (7.8)
	The PI can improve knowledge, satisfaction, medication adherence and/or quality of life OR the PI can prevent harm that does not require monitoring/treatment	Minor	926 (38.2)
	The PI can prevent harm that requires further monitoring/treatment but does not lead to or does not extend a hospital stay	Moderate	407 (16.8)
	The PI can prevent harm which causes or lengthens a hospital stay OR causes permanent disability or handicap	Major	787 (32.5)
	The PI can prevent an accident that potentially causes the need for intensive care or the death of the patient	Avoids fatality	5 (0.2)
	The available information does not allow the evaluation of clinical impact	Undetermined	97 (4.0)
Economic	The PI increases the cost of health care	Increase in cost	7 (0.3)
	The PI does not change the cost of health care	No change	787 (32.5)
	The PI decreases the cost of health care	Decrease in cost	1528 (63.2)
	The available information does not allow the evaluation of economic impact	Undetermined	97 (4.0)
Organisational	The PI reduces the quality of care	Negative	0
	The PI does not change the quality of care	No change	27 (1.1)
	The PI increases the quality of care	Positive	2367 (97.9)
	The available information does not allow the evaluation of organisational impact	Undetermined	25 (1.0)

*PI* Pharmacist intervention

## Discussion

This study assessed pharmacist-led activities and the potential outcomes of clinical activities in general practice. The findings of this study indicate that general practice pharmacists can provide a range of activities that has the potential to benefit patients, decrease healthcare costs for the government, and improve the quality of care. Our findings indicated that 59% of pharmacists’ activities were related to quality use of medicines. These activities included medication management services, such as medication reviews, transitions of care, and asthma care; medication safety initiatives such as clinical audits and POCT; and providing education to patients and general practice staff. Medication reviews were reported previously as the primary function of general practice pharmacists in Australia and other countries [9, 31–34]. In this study, most of the pharmacists’ recommendations were accepted by GPs and this is consistent with the literature for general practice pharmacists in Australia [20].

One of the roles of general practice-based pharmacists is to improve the quality and safety of prescribing through mechanisms such as practice-based audit and improvement cycles [9, 35]. Pharmacist-led clinical audits, POCT, and updating medical records can improve patient safety and quality of care in the general practice setting. Information provision to general practice staff and patients was also emphasised in this study. Providing education on medicines, devices, and lifestyle modifications (e.g. diabetes education and smoking cessation) were other activities that pharmacists conducted to benefit patients. Patient education, medication management and communication have been identified as key components to improve patient care [36]. Findings of this study have also shown the potential for collaborative activities between the general practice pharmacist and community pharmacy to benefit patients e.g. referring patients for community pharmacy services such as dose aid administration or sleep apnoea tests, delivering opioid maintenance treatment to patients, obtaining medicine availability information for GPs and patients, and solving queries related to medicine adherence or prescriptions.

The CLEO tool was modified to assess the potential clinical, economic, and organisational impact of pharmacist-led clinical activities in general practice [26]. Around 50% of pharmacist-led clinical activities had the potential to cause moderate and major positive clinical impact for patients (i.e., potentially reducing the risk of prolonged hospitalisation, or permanent disability in some cases), and around 63% of activities had the potential to decrease healthcare costs. In Australia, medication-related problems are estimated to cause at least 250,000 annual presentations to emergency departments and unplanned hospital admissions [37]. Furthermore, medication-related problems cost around AU$1.4 billion per annum to the Australian healthcare system [37]. Our findings suggest that general practice pharmacists could improve patient safety and reduce healthcare costs. This finding is consistent with international studies that have reported general practice pharmacists’ outcomes related to improving the quality and safety of medicines use [38–41].

This study has revealed that almost all the pharmacist-led activities contributed to improving the quality of care in general practice, as per the assessment with the CLEO tool [26]. Pharmacists’ contributions in saving time for GPs, service development, teamwork with other general practice members, and continuity of care were emphasised in a positive organisational impact. Pharmacists also supported GPs to generate income through contribution to MBS claimable items. This finding is consistent with previous studies conducted in Australia and Ireland [19, 23, 39]. The findings may be helpful in informing the development of a funding model for this role in the future.

### Strengths and limitations

This prospective observational study provided an overview of general practice pharmacist-led activities, as well as how pharmacists’ activities can contribute to targeting the prevention and resolution of medication-related problems. The study design allowed the longitudinal collection of information relating to multiple outcomes of general practice pharmacists. However, several limitations should be noted. This was a pragmatic observational study that did not include a control group. Furthermore, all general practices that employed a pharmacist in this study were located in the ACT, reflecting one of the states/territories in Australia. This may limit the generalisability of the results as other states or countries have different contextual factors. Pharmacists’ skillset and needs of the individual practice may have had an impact on the number of activities reported by each of the pharmacists. An independent panel was not involved, and GPs or other health professionals in general practice were not included in the panel when assessing the potential impact of pharmacist-led clinical activities. Thus, there is a potential for inherent subjectivity and bias in coding the activities and grading pharmacist-led clinical activities. The activities were self-reported by pharmacists, with the possibility for bias.

## Conclusion

Based on an adaptation of the CLEO tool, pharmacists’ activities had an overall positive impact in general practices, and resulted in improved clinical outcomes for patients, reduced healthcare costs, saved time for GPs, and improved continuity of care and teamwork in general practice. The study’s findings, while positive, require confirmation in a randomised controlled trial.

## Supplementary Information

Below is the link to the electronic supplementary material.

Supplementary file1 (DOCX 20 KB)
