# Supplementary material for: Evaluating the potential outcomes of pharmacist-led activities in the Australian general practice setting: a prospective observational study
Source: Int J Clin Pharm. 2023 Jun 3;45(4):980–8. doi: 10.1007/s11096-023-01604-x (PMC10239215; doi:10.1007/s11096-023-01604-x)
Supplement: Supplementary file 1 — Supplementary file1 (DOCX 20 KB) [file 11096_2023_1604_MOESM1_ESM.docx]

Supplemental file 1: Definitions for major codes

| Major code | Definition |
| --- | --- |
| Medication review | Conducting a structured evaluation of a patient’s medicines with the aim of optimising medicine use, minimising risk, and improving health outcomes. This includes identifying actual or potential drug-related problems, recommending interventions and the necessary follow-up actions in agreement with the patient. |
| Clinical audit | Being involved in any aspect of the clinical audit cycle. This comprises defining the criteria and setting the standards, conducting a search of the relevant electronic patient databases, and then reviewing the identified patients’ clinical notes to identify opportunities for improvement. The collaboration of the pharmacist with the GP, other healthcare professionals and the patient or carer, as appropriate, to suggest and implement recommendations for the patients identified. This also includes feedback to stakeholders on the broader outcomes of the clinical audit. |
| Antimicrobial stewardship | Conducting activities that encourage optimal antimicrobial prescribing and use. |
| Asthma | Conducting activities that aim to improve asthma management. |
| Transition of care | Conducting activities that support the coordination and continuity of health care during a movement of a patient from one healthcare setting to another. |
| Providing education | Educating general practice staff and patients. This includes medication-related queries from staff members or patients. |
| Diabetes education | Conducting activities that aim to improve diabetes management through pharmacological and non-pharmacological interventions, including as a credentialed diabetes educator (CDE). |
| Updating medical records | Conducting activities related to updating patients’ socio-demographic details, allergy status and adverse drug reactions. |
| Smoking cessation | Conducting activities that support smoking cessation through pharmacological and non-pharmacological interventions. |
| Point-of-care-testing | Conducting monitoring or diagnostic tests on a patient where the results are available without the need for laboratory analysis. |
| Vaccination | Administering or preparing vaccines. Logistical planning for immunisation. Consenting patients. |
| Collaboration with external service/healthcare providers | Collaborating with external health service providers or professionals e.g. community pharmacies, residential aged care facilities (RACFs), hospitals, community outreach services and other healthcare professionals (except where activities categorised under transition of care) |
| Service development | Supporting the development of a service. This includes planning, meetings, training or self-education, and booking appointments. |
| Other | Conducting activities that do not belong to any of the above categories. |

Supplemental file 2: Modified CLEO tool to evaluate general practice pharmacist-led activities

| **CLINICAL IMPACT** | | |
| --- | --- | --- |
| Score | Impact | Definition: The clinical impact is evaluated according to the most likely case expected, not the worst/best case |
| -1C | Negative | The PI can lead to adverse outcomes on clinical status, knowledge, satisfaction, patient adherence and/or quality of life of the patient. |
| OC | Null | The PI can have no influence on the patient regarding the clinical status, knowledge, satisfaction, patient adherence and or quality of life of the patient. |
| 1C | Minor | The PI can improve knowledge, satisfaction, medication adherence and/or quality of life OR the PI can prevent harm that does not require monitoring/treatment. |
| 2C | Moderate | The PI can prevent harm that requires further monitoring/treatment but does not lead to or does not extend a hospital stay. |
| 3C | Major | The PI can prevent harm which causes or lengthens a hospital stay OR causes permanent disability or handicap. |
| 4C | Avoids Fatality | The PI can prevent an accident that potentially causes the need for intensive care or the death of the patient. |
| UND | Undetermined | The available information does not allow the evaluation of clinical impact. |
| • The clinical impact was evaluated in terms of the benefit to the patient.  • Harm: alteration of physical and mental capacities arising from an accident or illness.  • Quality of life: physical function (autonomy, physical abilities, capacity to perform the tasks of daily life etc.), psychological (anxiety, depression, emotions etc.), social (relative to family environment, friends or professional contacts, engaging in personal relationships, participation in social and leisure activities etc.) and somatic (symptoms related to the disease).  • Monitoring: monitoring clinically relevant (physiological or psychological) variables, and/or biological parameters.  • Treatment: changing therapy or adding an additional medical/surgical treatment. | | |
| **ECONOMIC IMPACT** | | |
| Score | Impact | Definition |
| -1E | Increase in cost | The PI increases the cost of health care. |
| 0E | No change | The PI does not change the cost of health care. |
| 1E | Decrease in cost | The PI decreases the cost of health care. |
| UND | Undetermined | The available information does not allow the evaluation of economic impact. |
| • The cost of health care is based on the cost for the government: the cost of the drugs (eg. Pharmaceutical Benefits Scheme), and hospital presentations. | | |
| **ORGANISATIONAL IMPACT** | | |
| Score | Impact | Definition |
| -1O | Negative | The PI reduces the quality of care. |
| 0O | Null | The PI does not change the quality of care. |
| 1O | Positive | The PI increases the quality of care. |
| UND | Undetermined | The available information does not allow the evaluation of organisational impact. |
| • The organisational impact is coded in terms of the overall impact on the quality of the care process from the perspective of the health care providers (e.g. time-saving; improved security, knowledge or job satisfaction for nursing staff; facilitating tasks or teamwork, continuity of care etc.) | | |

PI - Pharmacist’s intervention

Supplemental file 3: Examples of case scenarios illustrating the impact of pharmacists’ clinical activities (CL = Clinical impact, E = Economic impact, O = Organisational impact)

| Case scenarios: examples | Impact |
| --- | --- |
| Pharmacist ceased esomeprazole 40 mg for a patient and their gastro-oesophageal reflux disease (GORD) symptoms returned. | CL=-1, E=0, O=0 |
| Pharmacist ceased isosorbide mononitrate for a patient over 65 years due to side effects, with no recommendation about a replacement therapy or monitoring the condition. | CL=-1, E=-1, O=0 |
| A patient with congestive cardiac failure was prescribed bisoprolol (beta blocker); however, the patient was not prescribed an angiotensin converting enzyme inhibitor (ACEI). Pharmacist recommended prescribing an ACEI for this patient. As the patient was under the care of the cardiologist, this recommendation was not accepted by the GP. | CL=0, E=0, O=1 |
| Pharmacist provided education on how to use a blood glucose monitor and modify the lifestyle of a patient who was newly-diagnosed with diabetes. | CL=1, E=1, O=1 |
| Pharmacist recommended a pill dispenser to facilitate the handling of medications out of the Webster pack for a diabetic patient over 65 years due to dexterity. Pharmacist provided education to avoid turmeric as it has a potential interaction with apixaban and metformin, and to check over-the-counter products with the community pharmacist before buying, to reduce the risk of medication interactions. | CL=2, E=1, O=1 |
| Pharmacist reconciled medicines for a patient with renal failure who was recently discharged from the hospital. Pharmacist updated pregabalin, calcium, and vitamin D dosages and advised the correct medicine list of patient to the community pharmacy. | CL=2, E=1, O=1 |
| A patient’s blood pressure was elevated due to poor adherence to prescribed medicines because of ADRs (flushing, tingling, headache). Pharmacist identified that ADRs were probably related to combined amlodipine, valsartan and hydrochlorothiazide. Pharmacist recommended replacing the combination with telmisartan. Pharmacist also updated the ADRs for this patient. This recommendation was accepted by the GP. | CL=3, E=1, O=1 |
| Pharmacist corrected the dry powdered inhaler technique for a patient who had exacerbation of asthma due to incorrect inhaler technique and poor medicine adherence. Pharmacist educated the patient on how to use the inhaler correctly, how to supplement with short-acting beta-agonists if required, and the importance of using their preventer regularly. Pharmacist also updated the allergy status and ADR for the patient. | CL=3, E=1, O=1 |
| Pharmacist identified a patient who had atrial fibrillation and high stroke risk but no prescribed anticoagulant by conducting a clinical audit. Pharmacist recommended an anticoagulant for this patient and followed-up. | CL=4, E=1, O=1 |
| Pharmacist reconciled medicines for a patient discharged from the hospital; identified and corrected discrepancies in the medicine list; corrected warfarin administration and improved adherence with unstable international normalised ratios (INR), and arranged dose administration aid service by liaising with the community pharmacy. | CL=4, E=1, O=1 |
